# Psychiatric Manifestations in an Adolescent With Voltage-Gated Potassium Channels (VGKC) Autoimmune Encephalitis: A Case Report

**DOI:** 10.7759/cureus.39960

**Published:** 2023-06-04

**Authors:** Safa F Hasan, Lubna Lutfi, Mahmood Shukur, Shokry Alemam, Hanan Esmaeel, Faisal Nawaz

**Affiliations:** 1 Department of Psychiatry, Al Amal Psychiatric Hospital, Dubai, ARE

**Keywords:** child and adolescent psychiatry, autoimmune encephalopathy, adolescent medicine, neuro-psychiatric, voltage-gated potassium channel autoimmune encephalitis

## Abstract

Autoimmune encephalitis (AE) is a newly defined group of disorders characterized by psychiatric symptoms such as psychosis and manic or hypomanic symptoms, with or without neurological symptoms. The most common neurological symptoms include seizures, altered mental status, autonomic disability, disorientation, and movement disorders. Our case report describes a type of AE caused by circulating autoantibodies against voltage-gated potassium channels (VGKC), which has not been reported before in the United Arab Emirates. This case report describes the psychiatric manifestations in a 17-year-old female with AE. It aims to shed light on the rare presentations of AE, discuss the various causes and management in greater depth, and the importance of suspecting and diagnosing AE early in the illness course. This rare case highlights the need for further research on the underlying biological, psychological, and social risk factors for developing AE in this region, and to direct further attention to developing early-intervention strategies in the vulnerable patient population.

## Introduction

Autoimmune encephalitis (AE) is a newly described group of disorders; it was believed to be part of the paraneoplastic disorder until the early 2000s when it was noted to be associated with different autoantibodies [1-3]. AE is caused by circulating autoantibodies targeting the synaptic or neuronal cell surface antigens, resulting in inflammation of the brain parenchyma. It is a rare disease with an estimated prevalence rate of 13.7/100,000 [2,4]. AE is characterized by psychiatric symptoms such as psychosis and manic or hypomanic symptoms, with or without neurological symptoms. The most common neurological symptoms include seizures, altered mental status, autonomic disability, disorientation, and movement disorders [3,5,6].

The various presentations of the disease make it a challenge to diagnose. Studies suggest common red flags that can predict the disease, including neurological symptoms and sensitivity to antipsychotics [5]. However, these red flags are vague, and AE can start with isolated psychiatric symptoms [7]. In addition, newer studies suggest an association between the affected part of the brain and the type of receptors with the presenting symptoms [1]. The most common neural or synaptic receptors are the N-methyl-D-aspartate receptor (NMDAR) and the leucine-rich glioma inactivated 1 (Lgi1). Rare autoantibodies or seronegative antibodies cause a significant number of AE cases; seronegative antibodies can be due to T-cell mediated reaction or another yet-to-be-identified antibody [1,2]. Autoimmune voltage-gated potassium channels (VGKC) encephalitis is a relatively common subtype of AE and is commonly associated with other autoimmune disorders or paraneoplastic phenomena [8]. Antibodies against VGKC were noted to be part of different neuronal hyper-excitability disorders such as Issacs’ syndrome, Morvan's syndrome, and limbic encephalitis [9,10]. Anti-VGKC antibodies target the VGKC complex, specifically the cell-surface antigens making the complex, not VGKC itself [9].

Early diagnosis and treatment are critical as the proper treatment will improve the patient outcomes significantly, while using antipsychotics in AE can result in life-threatening side effects of medications, including extrapyramidal symptoms and neuroleptic malignant syndrome [5,11]. This case report describes the psychiatric manifestations in a 17-year-old female with AE. It aims to shed light on the rare presentations of AE, discuss the various causes and management in greater depth, and highlight the importance of suspecting and diagnosing AE early in the illness course.

## Case presentation

A 17-year-old female, who had no past medical or psychiatric history, presented to the emergency room complaining of fever, headache, sore throat, and lower back pain of two-week duration. Furthermore, her family noticed a decrease in her concentration level and poor sleep, in addition to behavioral and personality changes, which they originally attributed to academic stressors. However, it became concerning when the patient developed irritability and had episodes of aggressiveness, impaired memory, and disorientation to persons, often not recognizing members of her own family. The patient was admitted to the hospital; lumbar puncture (LP) and magnetic resonance imaging (MRI) were performed upon admission which revealed no significant findings except for nonspecific changes in the frontal lobe white matter. Given the concern for viral encephalitis, acyclovir and ceftriaxone were started intravenously.

Following the resolution of her physical symptoms (headache, blurred vision, and myoclonic seizures), the patient was discharged 10 days after her admission. One day post discharge, she developed two attacks of myoclonic seizures lasting for a few minutes each and was re-hospitalized for further assessment. The psychiatry team was consulted due to the suspicion of psychogenic seizures and they recommended keeping the patient under the care of the neurology team and to repeat the LP to examine the autoimmune profile in CSF, as excluding viral and bacterial infections was not enough and did not explain the sudden onset of the symptoms and the episodic abnormal behaviors which the patient couldn’t remember in between the attacks.

During the second admission, the patient experienced five attacks of the same reported seizure. Therefore, she was transferred to the ICU and administered loading doses of both phenytoin and midazolam. Despite having a seizure while the EEG was performed, it showed no focal lateralizing or epileptiform discharges. The seizures were controlled with IV phenytoin and sodium valproate. The patient’s psychiatric symptoms persisted after being shifted back to the ward. Furthermore, the psychiatric symptoms started with disorientation to the family members (her aunt and cousins) for a few minutes and were repeated with her mother later. Also, she had poor sleep, was suddenly religiously over-occupied, had abnormal behaviors in the form of abnormal prayers, disinhibited behaviors against her nature, paranoid delusions, delusions of grandiosity, olfactory hallucinations, elated mood, labile affect, increased pressure and volume of speech, decreased attention and concentration, and flight of ideas.

As the history of illness was highly suggestive of AE, the patient was started on IV methylprednisolone and IV immunoglobulin.

Serological testing was conducted and revealed high titers of the VGKC-complex (587 pmol/L) (Table 1). Alpha-amino-3-hydroxy-5-methyl-4-isoxazolepropionic acid receptor (AMPA), N-methyl-D-aspartic acid (NMDA), and thyroid peroxidase antibody tests were all negative. Abdominal ultrasonography was performed and revealed normal findings. All remaining electrolyte levels were within the normal range and the patient was hemodynamically stable.

**Table 1:** Serological screen on day 1 of admission. Caspr2: contactin-associated protein-like 2; Lgi1: leucine-rich glioma inactivated 1

Test	Value
Voltage-gated Potassium Channel Antibodies	587+ pmol/L
Caspr2 Antibodies	Not detectable
Lgi1 Antibodies	Not detectable
Alpha-amino-3-hydroxy-5- methyl-4-isoxazole propionic acid receptor (AMPA) Antibodies	Not detectable
N-methyl-D-aspartic acid (NMDA) Antibodies	Not detectable
Thyroid Peroxidase Antibodies	Not detectable

**Figure 1:**
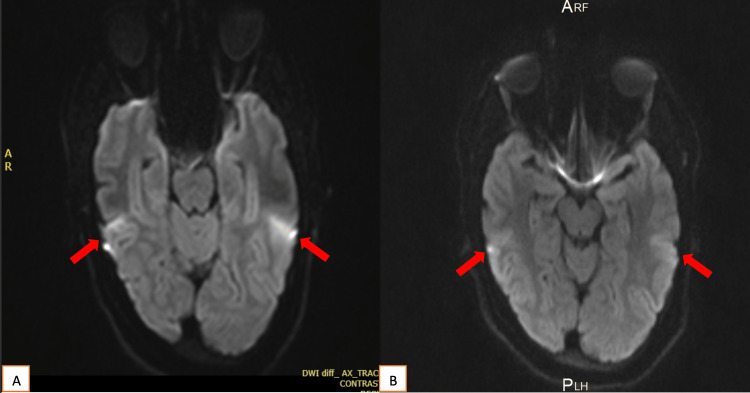
(A) Initial MRI scan; (B) MRI scan after one week of treatment

The patient’s mental status drastically improved after initiating IV methylprednisolone therapy and IV immunoglobulin (IVIG) for eight days. The result of serologial testing on day eight of treatment is given in Table 1. A comparison of iinital MRI with that taken one week after start of treatment is given in Figure 1. She was discharged after three weeks of hospitalization on phenytoin 100 mg, sodium valproate 500 mg twice daily, and prednisolone 60 mg once daily. She came for follow-up after two weeks with marked behavioral improvement and no more reported seizures. Her phenytoin dose was reduced to once daily and discontinued one week after discharge. She was advised to continue prednisolone 60 mg and Depakine Chrono 500 mg for one more month.

**Table 2:** Serological screen on day 8 of treatment.

Test	Value
Voltage-gated Potassium Channel Antibodies	273 pmol/L

On further follow-up at one and three months, the patient continued her improvement, euthymic mood, regular refreshing sleep, good attention and concentration, logic, and goal-directed thinking. There was no evidence of ongoing delusions and hallucinations, and the family denied any further seizures, or headaches, and reported normal attention and concentration.

## Discussion

This case report describes a type of AE caused by circulating autoantibodies against VGKC, which has not been reported before in the United Arab Emirates. The VGKC autoantibodies are known to cause limbic encephalitis, a nonparaneoplastic form of encephalitis [12].

As mentioned earlier, AE can be associated with different autoantibodies, each of which presents differently. Most common autoantibodies are neural or synaptic receptors such as NMDAR, Lgi1, and GABABR, while many cases are caused by rare autoantibodies or other seronegative antibodies [1,2]. AE usually presents with neurological symptoms, psychiatric manifestations, and a history of generalized illness [3,5,6]. For example, NMDAR antibodies could initially present with headache, fever, upper-respiratory-tract symptoms, and gastrointestinal symptoms such as nausea, vomiting, and diarrhea. It then proceeds to psychiatric manifestations of psychosis and manic or hypomanic symptoms [13].

However, it is more complicated in real-life scenarios because there is no clear history. For example, a case series of 20 patients with AE caused by GABAB receptors revealed that 17/20 presented with memory loss, confusion, hallucinations, personality change, and seizure [14]. In comparison, movement disorders such as dystonia and chorea were described in NMDAR and D2R-associated autoimmune AE [15]. In addition, status epilepticus was expressed in both GABABR and NMDAR [15].

Furthermore, VGKC causes limbic encephalitis (LE) that presents as memory loss, seizures, and psychiatric manifestations such as changes in affect and behavior [12]. A systematic review of 60 cases published in 2013 revealed that most patients (58/60) present with memory impairment, 51/60 developed seizures, and 20/60 showed a disturbance in affect [16]. In 2009, a case series of seven cases of VGKC LE published in Scotland showed that all seven patients presented with sub-acute seizures and anterograde memory loss [17]. In 2015, a case report also described worsening confusion and seizures [18].

Furthermore, while we relied on a serum specimen for serology testing in our case, antibody testing in CSF specimens is usually more accurate as serum can give a higher rate of false negative results [15]. The VGKC antibody test is an immunoprecipitation mainly containing VGKCs, in addition to Lgi1, contactin-associated protein-like 2 (Caspr2), and other proteins [15]. Post-treatment antibody titers revealed a decrease in their levels of up to 90% in some cases [16]. The challenge we faced in our case, and typically faced in similar scenarios, is that the final diagnosis is reached after antibody testing, which usually takes time, leading to a delay in treatment. This delay can worsen the prognosis [19,20].

In our case, the patient was managed with both IVIG and IV steroids. Guidelines for AE management vary from first-line broadly working immunosuppressants (steroids, IVIG, and plasma exchange) to second-line specific agents targeting a specific antibody-mediated disease process (rituximab and cyclophosphamide) [21]. Studies showed full immune remission within 1-4 months in 9 patients who received first-line immunotherapy through plasma exchange, IVIG, and IV steroids followed by oral steroids [22]. Further research shows that 83% of subjects who received first-line therapy improved significantly [16].

This report helps us recognize the different manifestations of AE and the red flags early in the course, thus creating a better outcome.

## Conclusions

AE of the VGKC type can be challenging to diagnose due to its variable presentation and psychiatric manifestations that co-exist with neurological symptoms. This rare case highlights the need for further research on the underlying biological, psychological, and social risk factors for developing AE in this region, and to direct further attention to developing early-intervention strategies in the vulnerable patient population.
